# High D-glucose levels induce ACE2 expression via GLUT1 in human airway epithelial cell line Calu-3

**DOI:** 10.1186/s12860-022-00427-4

**Published:** 2022-07-15

**Authors:** Yoshitaka Wakabayashi, Shin Nakayama, Ai Yamamoto, Takatoshi Kitazawa

**Affiliations:** Department of Internal Medicine, Teikyo University School of Medicine, 2-11-1 Kaga, Itabashi-ku, Tokyo, 173-8606 Japan

**Keywords:** ACE2, SARS-CoV-2, COVID-19, Angiotensin converting enzyme 2, Glucose transporters

## Abstract

**Background:**

Severe acute respiratory syndrome coronavirus 2 (SARS-CoV-2) enters the host cell by binding to angiotensin-converting enzyme 2 (ACE2) receptors. ACE2 is expressed on human airway epithelial cells. Increased ACE2 expression may be associated with potentially high risk of COVID-19. However, the factors responsible for the regulation of ACE2 expression in human airway epithelial cells are unknown. Furthermore, hyperglycemia is a risk factor for poor disease prognosis.

**Results:**

In this study, we investigated the effects of D-glucose on ACE2 mRNA and protein expressions in Calu-3 bronchial submucosal cells. The cells were cultured in minimal essential medium containing different D-glucose concentrations. After 48 and 72 h of high D-glucose (1000 mg/dL) treatment, ACE2 mRNA expressions were significantly increased. ACE2 protein expressions were significantly increased after 24 h of high D-glucose treatment. ACE2 mRNA expression was enhanced by a D-glucose concentration of 550 mg/dL or more after 72 h of treatment. In addition, we investigated the role of glucose transporters (GLUTs) in Calu-3 cells. ACE2 mRNA and protein expressions were suppressed by the GLUT1 inhibitor BAY-876 in high D-glucose-treated Calu-3 cells. GLUT-1 siRNA was also used and ACE2 mRNA expressions were suppressed in high D-glucose-treated Calu-3 cells with GLUT-1 knockdown.

**Conclusions:**

This is the first report indicating that high D-glucose levels induced ACE2 expression via GLUT1 in bronchial submucosal cells in vitro. As hyperglycemia can be treated appropriately, these findings could help reduce the risk of worsening of coronavirus disease 2019.

**Supplementary Information:**

The online version contains supplementary material available at 10.1186/s12860-022-00427-4.

## Highlights

- SARS-CoV-2 enters the host cell by binding to its receptor, angiotensin-converting enzyme 2 (ACE2).
- ACE2 is expressed in many cell types, including bronchial epithelial cells.
- High D-glucose medium induced ACE2 expression in bronchial epithelial Calu-3 cells.
- Glucose transporter 1 (GLUT1) is expressed in Calu-3 cells, and GLUT1 inhibition decreased ACE2 expression.

**Supplementary Information:**

The online version contains supplementary material available at 10.1186/s12860-022-00427-4.

## Background

Coronavirus disease 2019 (COVID-19) is an infectious disease caused by severe acute respiratory syndrome coronavirus 2 (SARS-CoV-2) [1]. It has spread rapidly around the world and has been declared a pandemic by the World Health Organization [2]. The case fatality rate is approximately 4% worldwide. However, fatality rates vary greatly between countries [3]. COVID-19 presents with various clinical features, ranging from asymptomatic to severe acute respiratory syndrome, hyperinflammatory response, and thrombosis [1, 4, 5]. Furthermore, COVID-19 can cause acute respiratory distress syndrome and multiple organ failure [1]. Some comorbidities such as hypertension, hyperlipidemia diabetes, and bronchial asthma may also increase COVID-19 severity [1].

The presence of diabetes mellitus and individual degree of hyperglycemia are associated with COVID-19 severity and increased mortality [6]. One retrospective cohort study has shown that mean glucose levels in the first 10 days of admission are higher on an average among those who died than those who survived [7]. In addition, patients with COVID-19 and hyperglycemia have a greatly enhanced inflammatory cytokine release or cytokine storm syndrome, which causes acute respiratory distress syndrome and multiple organ failure [8]. These studies indicate that tight control of glucose levels may be a key to prevent severe COVID-19. However, the mechanisms of hyperglycemia-related COVID-19 exacerbation remain unknown.

Angiotensin-converting enzyme 2 (ACE2) is a carboxypeptidase that negatively regulates the renin-angiotensin system, which can induce vasodilation by cleaving a single residue from the inactive decapeptide angiotensin I to generate angiotensin1–9 and degrade angiotensin II [9]. Recent studies have shown that ACE2 also acts as a SARS-CoV-2 receptor [10]. Baker et al. reported that ACE2 expression increased when patients are on mechanical ventilation because of lung alveolar damage, indicating a potential mechanism causing higher COVID-19 mortality [11]. In addition, plasma ACE2 levels in patients with severe COVID-19 in the late phase, are higher than those in patients with influenza [12]. An in vivo study has shown that ACE2 protein levels in the lungs of diabetic mice are elevated compared with those in the lungs of non-diabetic mice [13]. Hyperglycemia is thought to increase SARS-CoV-2 entry in airway epithelial cells through glycation of ACE2 [14] leading to severe symptoms. Therefore, we hypothesized that hyperglycemia induces ACE2 expression in the lungs. In this study, we investigated the effects of hyperglycemia on ACE2 expression in bronchial submucosal cells in vitro.

## Results

### ACE2 mRNA expression levels in Calu-3 cells grown in high D-glucose medium

**Fig. 1:**
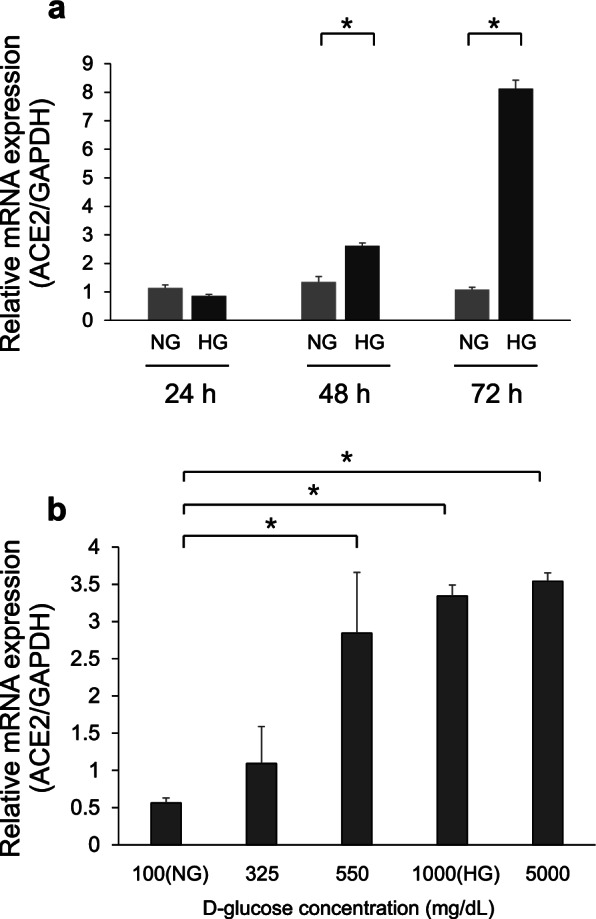
Real-time quantitative polymerase chain reaction analysis of angiotensin-converting enzyme 2 (ACE2) mRNA expression. **a** Calu-3 cells were treated with normal D-glucose (NG, 100 mg/dL) or high D-glucose (HG, 1000 mg/dL) for 24, 48, and 72 h. **b** Calu-3 cells were treated with 100–5000 mg/dL D-glucose concentrations. ACE2 gene expression was normalized relative to the expression of glyceraldehyde-3-phosphate dehydrogenase (GAPDH). Data indicated in the graph are mean fold increase ± SE over mean control value. Symbol (*) indicates statistical differences (*p* < 0.05). Data are presented as mean ± SD (*n* = 3)

We investigated the time dependency of ACE2 mRNA expression in Calu-3 cells; the cells were treated with normal D-glucose (NG) or high D-glucose (HG) for 24, 48, and 72 h. We used real-time quantitative polymerase chain reaction (qPCR) to determine ACE2 mRNA expression levels. ACE2 mRNA expression levels in HG-treated cells were not significantly different from those in NG-treated cells at 24 h. However, ACE2 mRNA expression in HG-treated cells was significantly elevated at 48 and 72 h (*p* < 0.05, Fig. 1a). Further, dose-titration of D-glucose was performed in Calu-3 cells at 72 h. The cells were treated with different D-glucose concentrations [100 (NG), 325, 550, 1000 (HG), and 5000 mg/dL]. Interestingly, ACE2 mRNA expression was significantly enhanced with 550, 1000, and 5000 mg/dL D-glucose treatments, but not with NG treatment (*p* < 0.05). Additionally, lower D-glucose concentrations did not enhance ACE2 mRNA expression (Fig. 1b).

### ACE2 protein levels in Calu-3 cells grown in HG medium

**Fig. 2:**
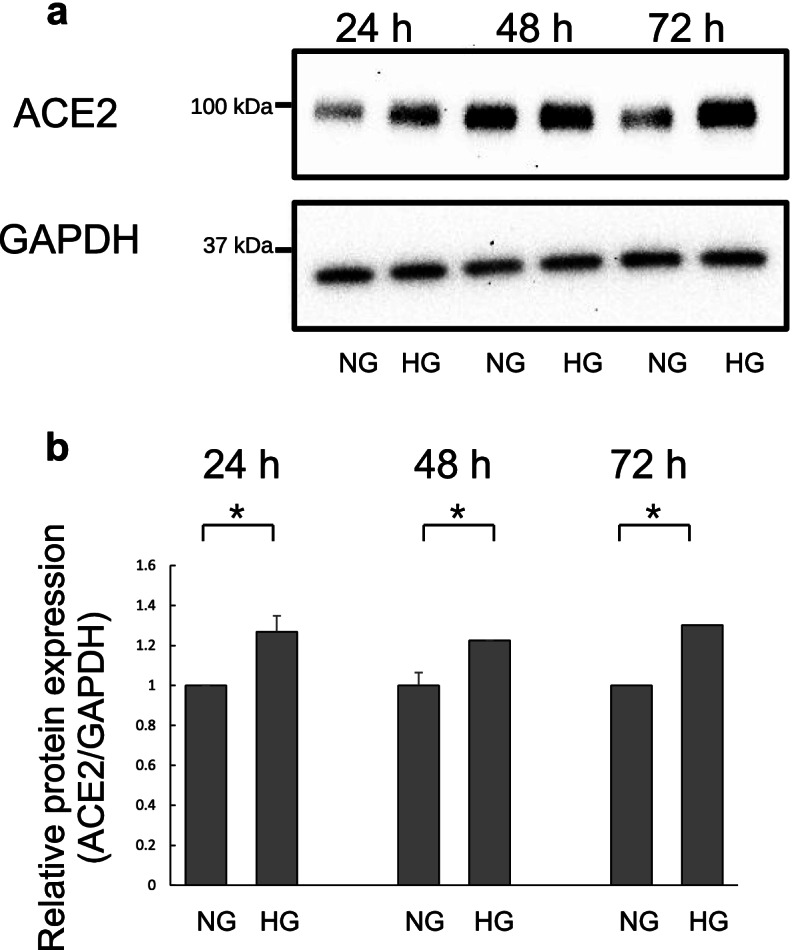
Western blot analysis of angiotensin-converting enzyme 2 (ACE2) expression in Calu-3 cells. **a** The cells were treated with normal D-glucose (NG) or high D-glucose (HG) for 24, 48, and 72 h. Whole cell lysates were collected and subjected to western blotting for ACE2 and glyceraldehyde-3-phosphate dehydrogenase (GAPDH). **b** The bar graph was generated by quantifying western blots from three independent experiments. Data are presented as mean ± SD (*n* = 6). Symbol (*) indicates statistical differences (*p* < 0.05)

To determine ACE2 protein expression levels after NG or HG treatment, we performed western blotting of cell lysates after 24, 48, and 72 h of treatment. Protein expression levels in HG-treated cells were elevated compared with those in NG-treated cells at 24, 48 and 72 h (Fig. 2a and b).

### Role of GLUT1 in ACE2 expression in Calu-3 cells

**Fig. 3:**
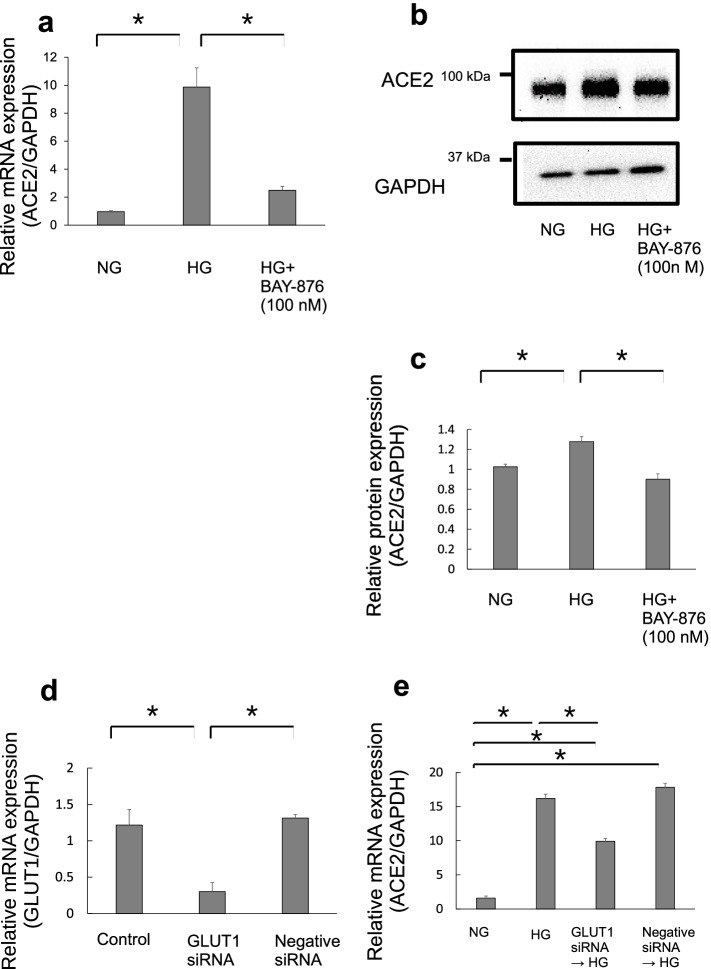
Inhibition of Glucose transporter (GLUT) gene in Calu-3 cells. The effects of GLUT1 inhibitor (BAY-876) on ACE2 expression were analyzed by real-time PCR (**a**) and western blotting (**b**, **c**) after 72 h of treatment. Data are presented as mean ± SD (*n* = 4). Symbol (*) indicates statistical differences (*p* < 0.05). **d** GLUT-1 mRNA was suppressed after GLUT-1 siRNA treatment for 24 h. **e** SiRNA-mediated knockdown of GLUT1 suppressed ACE2 mRNA in Calu-3 cells. Data are presented as mean ± SD (*n* = 3). Symbol (*) indicates statistical differences (*p* < 0.05)

Human bronchial cells express facultative glucose transporters (GLUTs) [15]. We used BAY-876, a GLUT1 inhibitor, to evaluate the role of GLUT1 in Calu-3 cells. The cells were treated with NG or HG media for 72 h. In addition, Calu-3 cells were treated with 100 nM BAY-876 for 72 h. ACE2 mRNA expression was measured using real-time PCR. Interestingly, ACE2 mRNA expression in the HG-treated group was significantly higher than that in the NG-treated group, and it was suppressed with 100 nM BAY-876 treatment (Fig. 3a). In addition, ACE2 protein expression levels, with or without BAY-876 treatment, were determined by western blotting. ACE2 protein expression was inhibited by BAY-876 in Calu-3 cells exposed to HG (Fig. 3b and c).

The effect of D-glucose after GLUT1 siRNA treatment on Calu-3 was also examined. Calu-3 cells were treated with medium or GLUT1 siRNA 24 h prior to the high glucose treatment. GLUT1 mRNA expression was suppressed after 24 h of GLUT1 siRNA treatment (Fig. 3d). Calu-3 cells were treated with HG for 72 h after GLUT1 siRNA treatment. ACE2 mRNA expression was measured using real-time PCR. ACE2 mRNA expression in the HG-treated group was significantly higher than that in the NG-treated group, and it was suppressed after GLUT1 siRNA treatment (Fig. 3e).

### Immunofluorescence staining for ACE2 in Calu-3 cells grown in HG medium

**Fig. 4:**
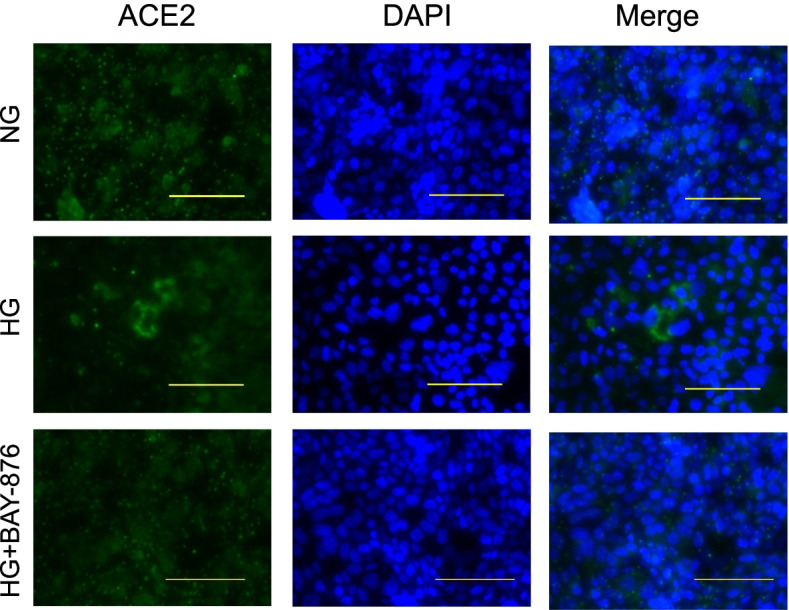
Immunofluorescence analysis of ACE2 expression in Calu-3 cells. The cells were treated with normal D-glucose (NG), high D-glucose (HG), or HG and 100 nM of glucose transporter 1 inhibitor (BAY-876) for 72 h. Green color indicates ACE2 expression, which was enhanced by HG and suppressed by BAY-876 in Calu-3 cells. Nuclei are stained in blue with DAPI. Scale bar, 100 μm

To evaluate ACE2 expression on the Calu-3 cell surface, we performed immunofluorescence staining after 72 h of NG or HG treatment. ACE2 staining intensity detected in HG-treated Calu-3 cells was higher than that in NG-treated Calu-3 cells. Notably, ACE2 staining intensity was lower in HG and BAY-876-treated Calu-3 cells than in HG-treated cells (Fig. 4).

### Cell viability and proliferation of HG-treated Calu-3 cells

**Fig. 5:**
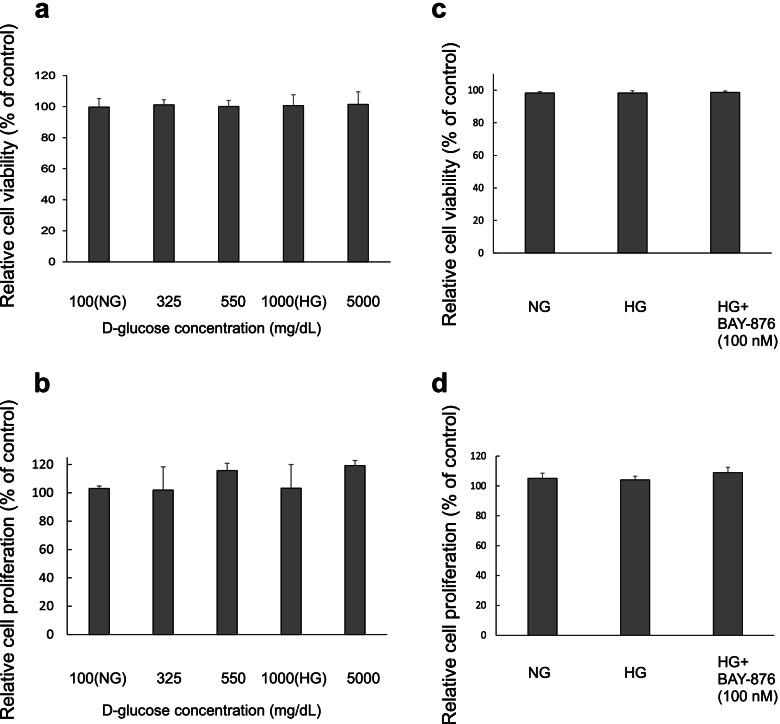
Analysis of Calu-3 cell viability and proliferation by lactate dehydrogenase assay and MTT assay. **a**, **c** Cell viability was measured by LDH assay after the cells were treated with different D-glucose concentrations and HG with BAY-876 for 72 h. **b**, **d** Cell proliferation was measured by MTT assay after the cells were treated with different D-glucose concentrations and HG with BAY-876 for 72 h. The figure shows that D-glucose levels and BAY-876 did not affect the viability of cultured cells. Data are represented as mean ± SD (*n* = 3 per group). NG, normal D-glucose; HG, high D-glucose

The cells were treated with different D-glucose concentrations [100 (NG), 325, 550, 1000 (HG), and 5000 mg/dL] and HG with 100 nM of BAY-876 for 72 h. LDH activity and the 3-(4,5-dimethylthiazol-2-yl)-2,5-diphenyl-2H-tetrazolium bromide (MTT) assay was measured to evaluate cell viability and proliferation. No statistically significant differences were observed between all groups. These results showed that high glucose medium up to 5000 mg/dL did not have cytotoxic and proliferative effects on Calu-3 cells (Fig. 5a-d).

## Discussion

We showed that HG levels enhanced ACE2 mRNA and protein expression levels in Calu-3 cells via GLUT1 in a dose- and time-dependent manner. Vuong Cat Khanh et al. already showed ACE2 expression in Calu-3 cells were enhanced under high glucose concentration [16]., and we found that GLUT1 is the receptor to enhance expression of ACE2.

SARS-CoV-2 enters host cells using viral surface transmembrane spike (S) glycoprotein [17]. The S glycoprotein is cleaved into S1 and S2 subunits [17]. As mentioned above, ACE2 serves as a SARS-CoV-2 receptor. The S1 subunit recognizes and binds to ACE2, and the S2 subunit is responsible for viral fusion and entry into host cells [17].

It is well known that ACE2 is expressed in enterocytes, renal tubules, gallbladder, cardiomyocytes, male reproductive cells, placental trophoblasts, ductal cells, eyes, and vasculature [18]. Moreover, ACE2 expression is lower in the lungs than in these tissues [19]. However, pulmonary ACE2 expression is important in the development of infectious disease and has received extensive attention in recent years, as ACE2 serves as a SARS-CoV-2 receptor [10]. ACE2 is expressed on the surface of human lung airway and alveolar epithelial cells [20], and ACE2 expression level is positively correlated with airway epithelial differentiation [21]. In addition, lung ACE2 expression is higher in people with comorbidities, such as cancer, hypertension, diabetes, and chronic obstructive lung disease, thereby increasing their susceptibility to COVID-19 [22]. However, the mechanism underlying the induction of ACE2 expression in the lungs remains unknown.

Calu-3 cells are derived from human bronchial submucosal glands and are generated from bronchial adenocarcinoma [23]. Calu-3 cell line is commonly used in SARS-CoV-2 studies because it reflects the properties of bronchial submucosal glands and secretes airway surface liquid, mucins, and other immunologically active substances [23].

High ACE2 expression has been thought to be a risk factor for COVID-19 severity. Ackermann et al. reported that lung ACE2 expression was higher in patients who died of COVID-19 than that in uninfected control subjects [5]. Kragstrup et al. also reported that elevated baseline plasma ACE2 in patients with COVID-9 was significantly associated with increased disease severity during the 28-day study period [24].

Furthermore, diabetes and hyperglycemia have also been considered as risk factors of severe COVID-19 [6]. In patients with COVID-19, higher serum C-reactive protein, interleukin-6, and LDH levels, which correlated with disease severity, were observed in the diabetic group with hyperglycemia than in the non-diabetic group [6, 25]. Another study suggested that hyperglycemia was the greatest risk factor for 14-day and 60-day in-hospital mortality among critically ill patients with COVID-19 [26]. It is worth noting that hyperglycemia has been observed not only in diabetic patients with COVID-19, but also in patients without any clinical history of diabetes mellitus. The use of corticosteroids in patients with COVID-19 who require oxygen therapy is recommended in clinical practice [27]. One of the side effects of corticosteroid therapy is increased blood glucose levels [28]. Therefore, hyperglycemia is often observed in patients undergoing corticosteroid treatment for COVID-19 at our facility. Uncontrolled glucose levels are considered a poor prognostic factor, and stringent control of glucose levels is important for the treatment of COVID-19 [29].

Lavrentyev et al. reported that ACE2 mRNA expression levels are upregulated in vascular muscle cells after 2 and 4 h of high glucose treatment [30]. Our results showed that, in the late phase of high glucose treatment, ACE2 expression in lung cells was enhanced compared with that in vascular muscle cells. This indicates the possibility that a longer time might be required to enhance ACE2 expression after D-glucose stimulation in vivo*,* and further in vivo research will be required.

D-glucose is transported across mammalian cell membranes through two types of receptors: facilitative GLUTs and sodium-glucose linked transporters [15]. Human airway epithelial cells primarily express GLUTs [15]. GLUTs are divided into three classes according to their sequence similarity. Class 1 consists of GLUT 1–4 and 14, which transport D-glucose [31]. Using real-time qPCR, we confirmed that GLUT1 was predominantly expressed in Calu-3 cells. The role of GLUTs in the airway epithelium is under investigation. However, they might play a key role in airway metabolism and regeneration. Furthermore, human immunodeficiency virus and malaria infections are regulated by GLUT1, but there is no research on the relationship between GLUT1 and SARS-CoV-2 infection [32, 33]. We used GLUT1 inhibitor BAY-876 to demonstrate that ACE2 expression is regulated by GLUT1. Thus, GLUT1 might play a crucial role in COVID-19 infection by controlling ACE2 expression; however, further investigation is needed.

It was previously reported that ACE2 expression is induced by cytotoxic stimulation in epithelial bronchial cells in vitro [34]. Hence, we needed to exclude the possibility that the cytotoxicity of high glucose levels reinforced ACE2 expression. Interestingly, LDH levels was not significantly changed in Calu-3 cells after 72 h of HG treatment compared to NG treatment. These results indicated that glucose levels up to 1000 mg/dL were not cytotoxic to Calu-3 cells, and ACE2 expression was enhanced by D-glucose itself, not by D-glucose cytotoxicity.

The limitation of our study is that these phenomena are only observed in vitro, and further in vivo studies are required to understand the correlation between ACE2 expression levels and hyperglycemia. In addition, we did not show a relationship between high glucose-induced high ACE2 expression and susceptibility to SARS-CoV-2.

Normally, fasting blood glucose levels in humans are maintained within a range of 4–6 mmol/L or 72–108 mg/dL [35]. MEM contained 100 mg/dL of D-glucose, and Calu-3 cells cultured in MEM were used as a control group in this study. Hyperglycemia is defined as a blood glucose level greater than 140 mg/dL [36]. In our study, ACE2 expression in cells treated with more than 550 mg/dL D-glucose was enhanced compared with that in the control group. When blood glucose in the human body is consistently at this high level, emergent diseases, such as diabetic ketoacidosis or a hyperglycemic hyperosmolar state develop [37]. However, topical D-glucose concentration in the airway and alveoli after corticosteroid use remains unknown. We need to confirm this result in vivo whether ACE2 expression in lung is enhanced with lower glucose level.

## Conclusions

We have demonstrated that high glucose levels induce ACE2 expression via GLUT1 in Calu-3 cells. These new findings suggest the possibility that hyperglycemia induces ACE2 expression in the lungs, which worsens COVID-19. Close monitoring and tight control of blood glucose levels by oral medication or insulin treatment might be important for preventing the clinical worsening of patients with COVID-19.

## Materials and methods

### Cell line and culture

Calu-3, a human submucosal gland cell line derived from bronchial adenocarcinoma, was purchased from Elabscience Biotechnology Inc. (USA). Calu-3 cells were cultured at 37 °C with 5% CO_2_ in MEM (Nacalai Tesque, Inc., Kyoto, Japan) with 10% fetal bovine serum (Hyclone, Logan, UT, USA), 100 IU/mL penicillin, and 100 μg/mL streptomycin (Nacalai Tesque, Inc.). The cells were stimulated with different D-glucose concentrations as follows: 100 mg/dL D-glucose (Nacalai Tesque, Inc.) in MEM (NG) and 1000 mg/dL D-glucose in MEM (HG). The medium was changed every alternate day. BAY-876 (Medchemexpress, Monmouth Junction, NJ, USA) was used to inhibit GLUT1.

### Total RNA extraction and cDNA synthesis

Calu-3 cells were seeded at a density of 4 × 10^5^ cells/well in 24-well plates. After 24 h, the medium was replaced with medium containing different D-glucose concentrations (100 and 1000 mg/dL) and cultured at 37 °C with 5% CO_2_ for 24, 48, and 72 h. Total RNA was extracted with 180 μL Tripure (Roche, Basel, Switzerland) from each well following manufacturer’s instruction. Total RNA was reverse transcribed to cDNA with ReverTra Ace® qPCR RT Master Mix (Toyobo Life Science, Osaka, Japan).

### Real-time qPCR

ACE2 gene expression in Calu-3 cells was examined by real-time qPCR. Glyceraldehyde-3-phosphate dehydrogenase (GAPDH) was used as an internal control. Real-time qPCR was performed on a Roche LightCycler 480 system (Roche Diagnostics) using THUNDERBIRD® SYBR® qPCR Mix (Toyobo Life Science). Reactions were performed in a final volume of 20 μL containing 1x THUNDERBIRD® SYBR® qPCR Mix, 0.3 μM of forward and 0.1 μM of reverse primers, 7.8 μL of distilled H_2_O, and 1 μL of the cDNA template. The primers used in this study were as follows: For human ACE2, forward, 5′-CCACTGCTCAACTACTTTGAGCC-3′ and reverse, 5′-CTTATCCTCACTTTGATGCT-3′; for GAPDH, forward, 5′-GAAGGTGAAGGTCGGAGTC-3′ and reverse, 5′-GAAGATGGTGATGGGATTTC-3′; for GLUT1, forward, 5′-AAGGTGATCGAGGAGTTCTACA-3′ and reverse, 5′- ATGCCCCCAACAGAAAAGATG-3′. The amplification conditions were as follows: denaturation at 95 °C for 5 min; 45 cycles of amplification (95 °C for 10 s, 60 °C for 10 s, and 72 °C for 10 s); and cooling at 50 °C for 30 s. Relative quantification of target gene expression was determined using LightCycler 480 software according to ΔΔCT-method.

### Western blotting

Lysate samples were isolated from Calu-3 cells using a radioimmunoprecipitation assay lysis buffer (Wako Pure Chemical Industries, Osaka, Japan). Total protein was fractionated using 10% sodium dodecyl sulfate-polyacrylamide gel electrophoresis and transferred to polyvinylidene fluoride membrane. After blocking with 5% bovine serum albumin in Tris-buffered saline/0.1% Tween-20 (TBST) at room temperature for 1 h, the membrane was incubated overnight at 4 °C with antibodies against ACE2 (1:1000; R&D Systems, Inc. Minneapolis, MN, USA) and GAPDH (1:2000, Santa Cruz Biotechnology, CA, USA). The membrane was washed three times with TBST and incubated with horseradish peroxidase anti-goat or anti-mouse secondary antibody (DakoCytomation, CA, USA) at 20 °C for 1 h. After washing three times with TBST, immunoreactive bands were detected using a ChemiDoc XRS+ Image System (Bio-Rad Laboratories, CA, USA). Protein bands were quantified using Image Lab software (Bio-Rad Laboratories).

### Immunofluorescence staining

Calu-3 cells were cultured on cover-glass slides for 72 h. The glass slides were washed three times with phosphate buffered saline (PBS), fixed with 4% paraformaldehyde (Nacalai Tesque, Inc.) for 15 min at room temperature, and rinsed with PBS. After blocking with PBS containing 5% bovine serum albumin for 30 min, fixed Calu-3 cells were incubated overnight at 4 °C with primary antibody mouse anti-human ACE2 (1:50; Santa Cruz Biotechnology). After rinsing with PBS, the slides were incubated with anti-mouse immunoglobulin G H&L (Alexa Fluor® 488; Abcam, MA, USA) in PBS. After rinsing with PBS, slides were stained with DAPI (Tokyo Chemical Industry, Tokyo, Japan) for 15 min at room temperature. The cover slides were mounted using Fluoromount (Diagnostic BioSystems, CA, USA), scanned, and photographed using a light microscope (Keyence BZ-X800, Keyence Corporation of America, NJ, USA).

### LDH activity assay

LDH activity was measured to determine the effect of D-glucose concentration on cell viability. Calu-3 cells were plated in 96-well plates (1 × 10^5^ cells/well) and incubated in MEM with different D-glucose concentrations ranging from 100 to 5000 mg/dL, and with BAY-876. After 72 h, cell viability was evaluated using the Cytotoxicity Detection Kit PLUS (Roche Diagnostics), according to the manufacturer’s protocol. Cells cultured in MEM were used as controls. The absorbance was measured at 490 nm, and cell viability was calculated.

### The 3-(4,5-dimethylthiazol-2-yl)-2,5-diphenyltetrazolium bromide (MTT) assay

Cell proliferation and viability were assessed using an MTT assay (Nacalai Tesque, Inc.). Calu-3 cells were plated in 96-well plates (1 × 10^5^ cells/well) and incubated in MEM with different D-glucose concentrations ranging from 100 to 5000 mg/dL, and inhibitors (BAY-876) for 72 h. Following instruction, 10 μl MTT solution was added, and the cells were cultured at 37 °C with 5% CO2 for 4 h. 100 μl solubilization solution was added, and the cells were cultured at 37 °C with 5% CO2 for 15 h. The absorbance was measured at 570 nm.

### RNA interference (siRNA)

The pre-designed Silencer™ Select Validated siRNA or Silencer™ Select siRNA negative control (Thermo Fisher Scientific, MA, USA) were transfected into Calu-3 cells using 10 nM of Lipofectamine™ RNAiMAX (Thermo Fisher Scientific) manufacturer’s instructions. 24 h after transfection, cells were treated with NG or HG for 72 h.

### Statistical analysis

Significant differences among samples were determined by one-way analysis of variance with post-hoc Tukey’s honest significant difference test or Student’s t-test with *p* < 0.05 indicating statistical significance. A minimum of three samples were tested in three independent experiments. All results are presented as mean ± standard error of the mean. Data analysis was performed using the SPSS software version 24.0 (IBM, Armonk, NY, USA).

## Supplementary Information

**Additional file 1.**

## Data Availability

All data analyzed in the current study are available from the corresponding author upon reasonable request.

## Abbreviations

ACE2: Angiotensin-Converting Enzyme 2
COVID-19: Coronavirus Disease 2019
GAPDH: Glyceraldehyde-3-Phosphate Dehydrogenase
GLUT: Glucose Transporter
HG: High D-Glucose
LDH: Lactate Dehydrogenase
MEM: Minimal Essential Medium
MTT: 3-(4,5-dimethylthiazol-2-yl)-2,5-diphenyl-2H-tetrazolium bromide
NG: Normal D-Glucose
qPCR: Quantitative Polymerase Chain Reaction
SARS-CoV-2: Severe Acute Respiratory Syndrome Coronavirus 2
