# Supplementary figures and images for: High D-glucose levels induce ACE2 expression via GLUT1 in human airway epithelial cell line Calu-3
Source: BMC Mol Cell Biol. 2022 Jul 15;23:29. doi: 10.1186/s12860-022-00427-4 (PMC9282902; doi:10.1186/s12860-022-00427-4)

24h 48h 72h NG HG

1

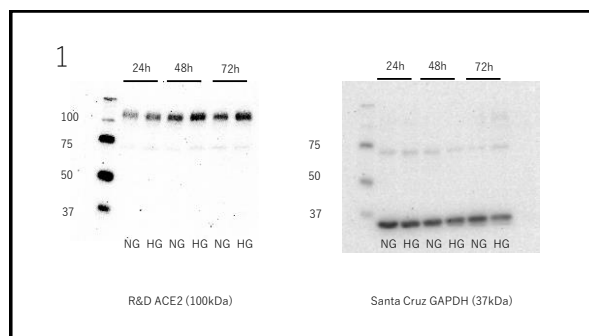

2

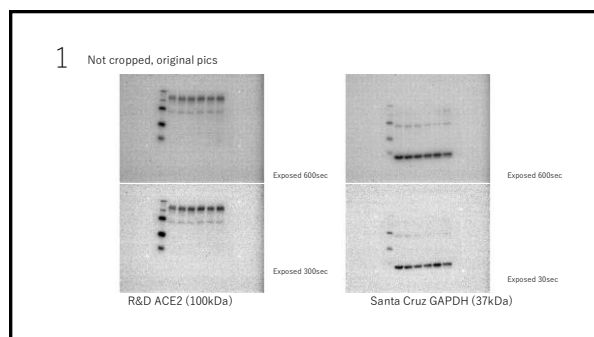

3

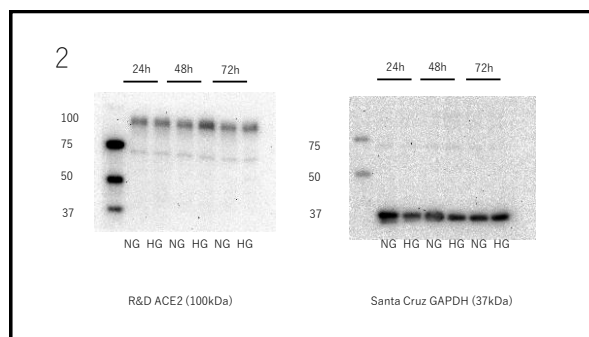

4

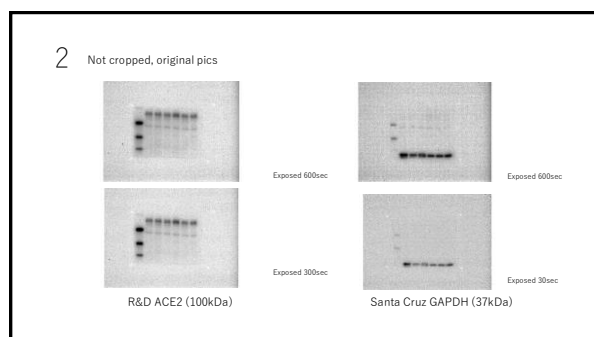

5

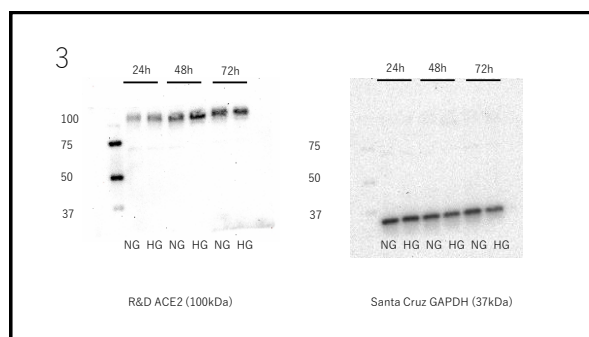

6

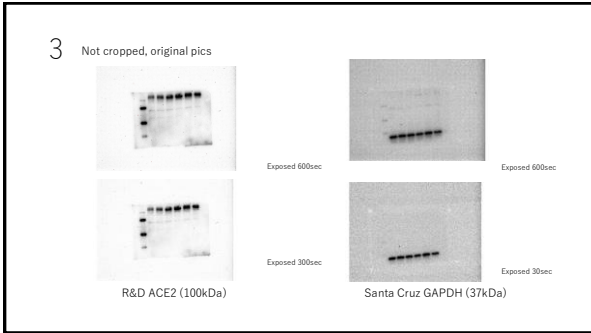

7

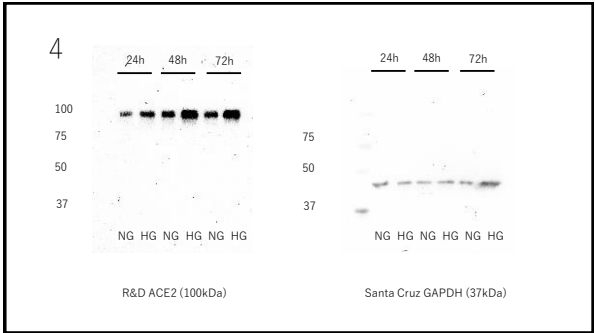

8

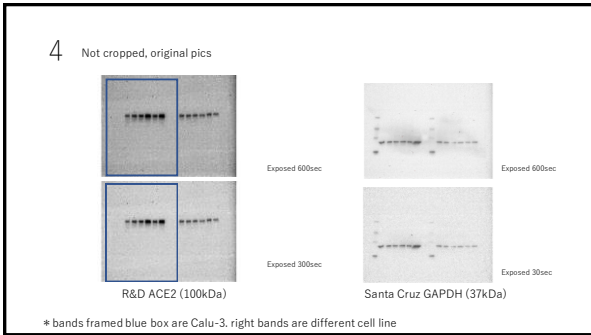

9

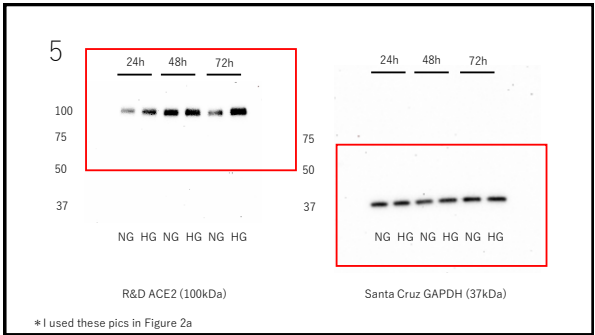

10

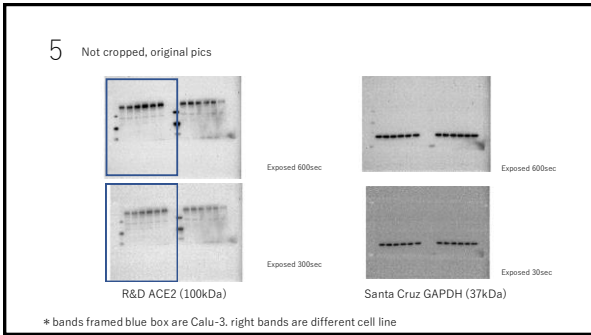

11

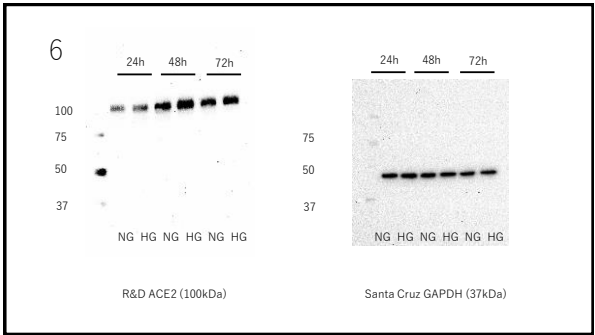

12

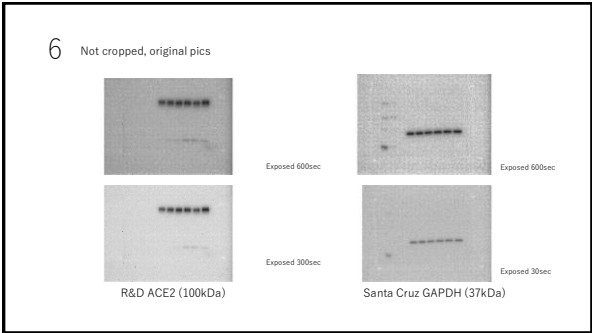

13

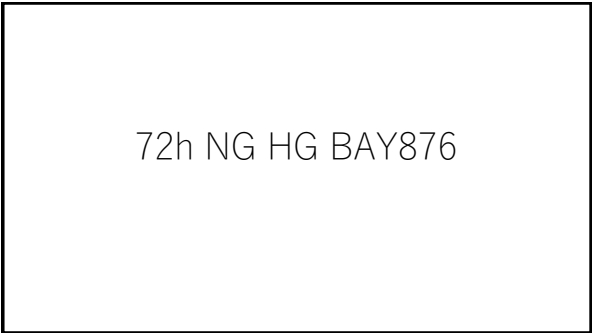

14

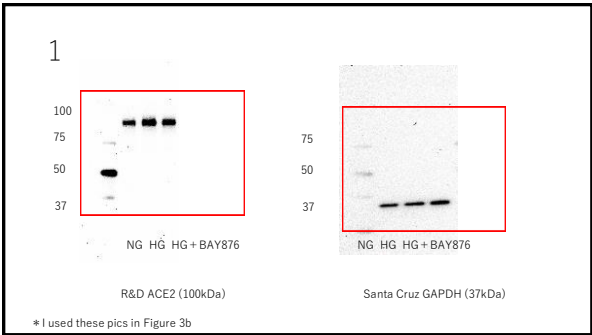

15

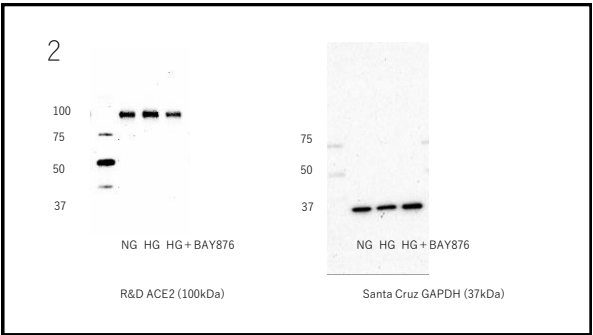

16

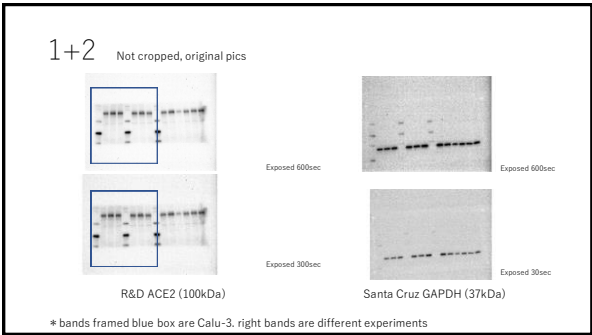

17

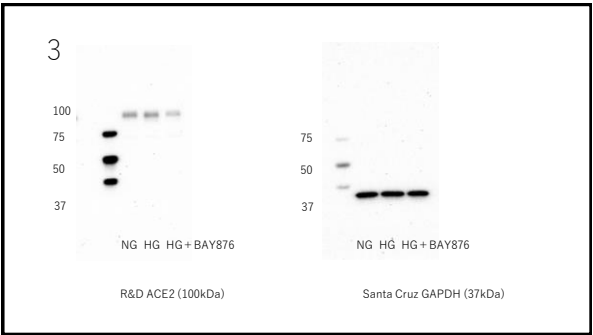

18

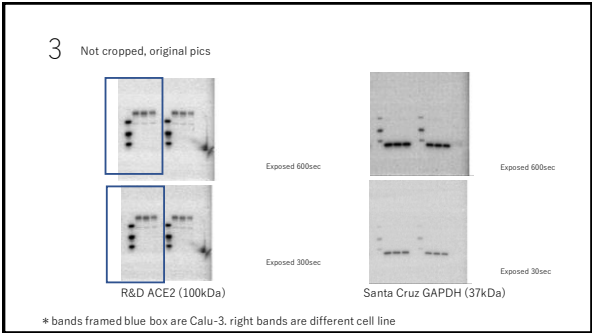

19

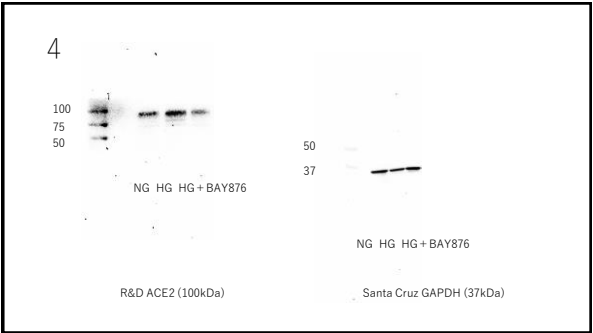

20

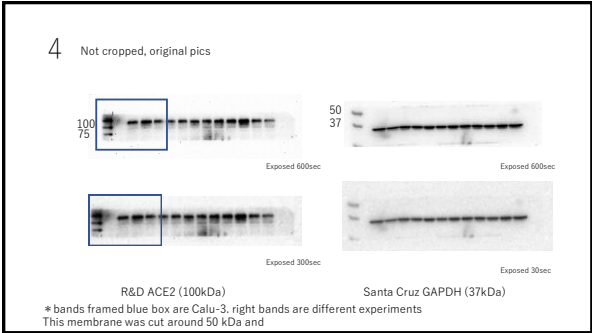

21

Supplement: Supplementary file 1 — Additional file 1. [file 12860_2022_427_MOESM1_ESM.pdf]
